# Transport Asymmetry of Novel Bi-Layer Hybrid Perfluorinated Membranes on the Base of MF-4SC Modified by Halloysite Nanotubes with Platinum

**DOI:** 10.3390/polym10040366

**Published:** 2018-03-25

**Authors:** Anatoly Filippov, Daria Petrova, Irina Falina, Natalia Kononenko, Evgenii Ivanov, Yuri Lvov, Vladimir Vinokurov

**Affiliations:** 1 Laboratory of functionalized aluminosilicate materials, Department of Physical and Colloid Chemistry, Gubkin University, Leninsky prospect 65-1, Moscow 119991, Russia; petrova.msu@gmail.com (D.P.); ivanov166@list.ru (E.I.); ylvov@coes.latech.edu (Y.L.); vinok_ac@mail.ru (V.V.); 2 Department of Higher Mathematics, Gubkin University, Leninsky prospect 65-1, Moscow 119991, Russia; 3 Chemistry Department, Lomonosov Moscow State University, Leninskiye Gory 1, Moscow 119991, Russia; 4 Department of Physical Chemistry, Kuban State University, Stavropol’skaya 149, Krasnodar 350040, Russia; irina_falina@mail.ru (I.F.); kononenk@chem.kubsu.ru (N.K.); 5 Institute for Micromanufacturing, Louisiana Tech University, 911 Hergot Ave., Ruston, LA 71272, USA

**Keywords:** hybrid membrane, perfluorinated sulfocationic membrane, halloysite, platinum nanoparticles, diffusion permeability, asymmetry of current–voltage characteristics, modeling transport through a bi-layer membrane

## Abstract

Three types of bi-layer hybrid nanocomposites on the base of perfluorinated cation-exchange membrane MF-4SC (Russian analogue of Nafion^®^-117) were synthesized and characterized. It was found that two membranes possess the noticeable asymmetry of the current–voltage curve (CVC) under changing their orientation towards the applied electric field, despite the absence of asymmetry of diffusion permeability. These phenomena were explained in the frame of the “fine-porous model” expanded for bi-layer membranes. A special procedure to calculate the real values of the diffusion layers thickness and the limiting current density was proposed. Due to asymmetry effects of the current voltage curves of bi-layer hybrid membranes on the base of MF-4SC, halloysite nanotubes and platinum nanoparticles, it is prospective to assemble membrane switches (membrane relays or diodes) with predictable transport properties, founded upon the theory developed here.

## 1. Introduction

Ion-exchange membranes are intensively used in fuel cells [1], as well as in electrolysis, dialysis, electrodialysis, and other membrane methods for obtaining, separating, and purifying various mixtures in sensors and transducers during gas transportation [2]. The most extensively applied materials for such membrane types are Nafion-117^®^, produced by DuPont de Nemours (Wilmington, DE, USA), and its analogues, such as MF-4SC (LTD Plastpolymer, Saint Petersburg, Russia) and Dow (Dow, Midland, MI, USA) [3,4]. Incorporation in polymer frame-varying modifiers influences physicochemical properties, for example, ion transport, mechanical properties, diffusion, and electroosmotic permeability. One of approaches to improve efficiency of transport properties, as well as reducing fuel cell cost, is use of cheap natural modifiers capable of forming a nanoporous membrane structure, which is necessary for ion transport process. In this regard, aluminosilicate nanotubes (halloysite) and their variations, which are ecologically safe, obtainable, and cheap [5], can be applied as a dopant. In addition, the halloysite structure has features that allow its modification [6]. Halloysite has a positively charged inner lumen and a negatively charged outer surface [6] that is used for intercalation and encapsulation tubes by various metals [7,8,9]. Nanotubes, coated or intercalated with metal and metal oxides (TiO_2_, Pd, Ni), are efficient mesoporous media for advanced catalysis [8,10,11]. The ability of halloysite to be mixed with various kinds of polymers (e.g., polysaccharides, polyacrylates, polyamides, epoxy, poly(vinyl chloride), polyethylene) has been described in many research studies [7,12,13]. Nanotubes form frameworks inside the polymer matrix, improving strength, and increasing elongation limits and porosity of membrane materials.

According to [14], incorporation of halloysite nanotubes modified by platinum in ion-exchange membranes leads to advanced properties of membrane used as a solid electrolyte in fuel cells. Using halloysite as a dopant influences asymmetry of diffusion permeability and current–voltage characteristics (CVC), decreases the numbers of water transport, and increases the selectivity of hybrid membranes, and their power characteristics in the membrane–electrode assembly of the fuel cell [15]. The theoretical base of the asymmetry effect for diffusion permeability of bi-layer membranes is established in our papers [16,17]. A search of effective solutions in the field of alternative energy sources is impossible without a detailed study of the fundamental principles of membrane processes.

The aim of the present paper is to investigate novel bi-layer membranes synthesized on the base of MF-4SC and halloysite nanotubes modified by Pt nanoparticles, to perform its characterization and to apply previously developed models, which will allow calculating asymmetry of membrane diffusion permeability and membrane CVC in order to reach the best quality for use in fuel cells and membrane switches (membrane relays or diodes).

## 2. Experiment

### 2.1. Materials and Instruments

Dehydrated halloysite nanotubes were obtained from Applied Minerals Inc., New York, NY, USA, solution of sulfopolymer of the MF-4SC in lithium form (7.2 wt % in dimethylformamide solution, with 0.98 mgeq/g exchange capacity) was purchased from Plastpolymer, Sankt-Peterburg, Russia.

For halloysite nanotubes modification following reagents were used: 3-aminopropyltriethoxysilane, APTES (99%, Sigma Aldrich, Saint Louis, MO, USA), hexachloroplatinic acid hexahydrate H_2_PtCl_6_·6H_2_O (99.9%, Sigma Aldrich), toluene (99.8%, Sigma Aldrich), sodium tetrahydridoborate NaBH_4_ (98%, Sigma Aldrich), and distilled water for rinsing.

Electron microscope JEM-2100 (JEOL, Tokyo, Japan) was applied for transmission electron microscopy (TEM) analyses at an accelerating voltage of 10 kV. Atomic force microscopy (AFM) studies were carried out by the scanning probe microscope SmartSPM^®^-1000 (AIST-NT, Novato, CA, USA) in semi-contact mode using fpN11 cantilever (beam length 130 µm, hardness 2.6–9.8 N/m, resonance frequency of 118–190 kHz, radius of curvature of the probe needles—10–25 nm).

### 2.2. Сlay Nanotubes Modification

Deposition of platinum nanoparticles onto the surface of the nanotubes was carried out by grafting NH_2_ groups on the outer surface of the halloysite nanotubes. For this purpose, we used APTES, halloysite nanotubes, and dry toluene. Nanotubes were diffused in concentrated APTES solution and left for 12 h with stirring. The obtained nanotubes were centrifuged, washed three times with toluene, and dried at 60 °C. The nanotubes modified by APTES were diffused in H_2_PtCl_6_∙6H_2_O solution in ultrasonic bath for 30 min followed by reduction by NaBH_4_. After the nanotubes were centrifuged, washed three times, and dried.

**Figure 1:**
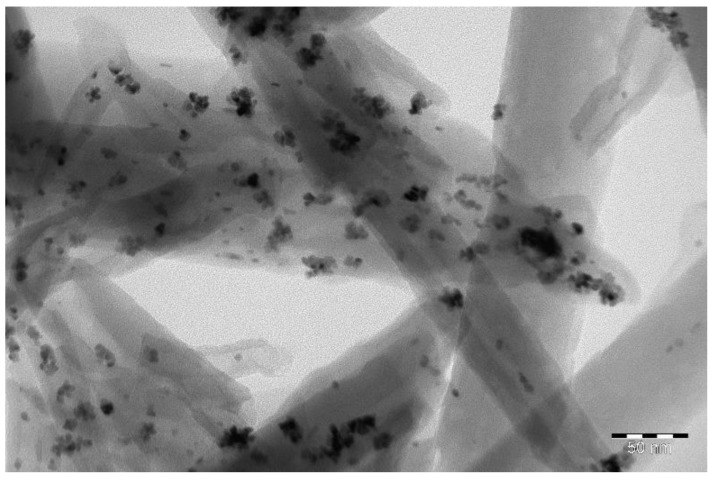
Electron micrography (TEM) of halloysite nanotubes modified over the outer surface by Pt nanoparticles.

**Figure 2:**
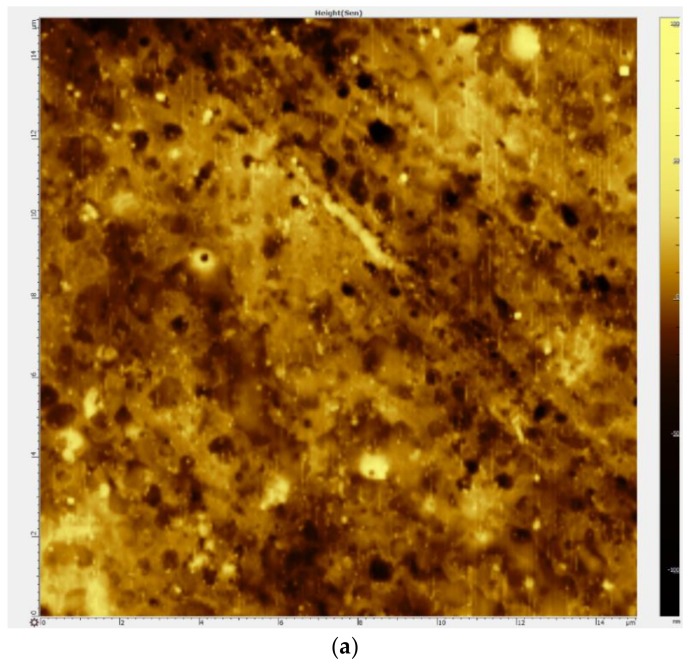

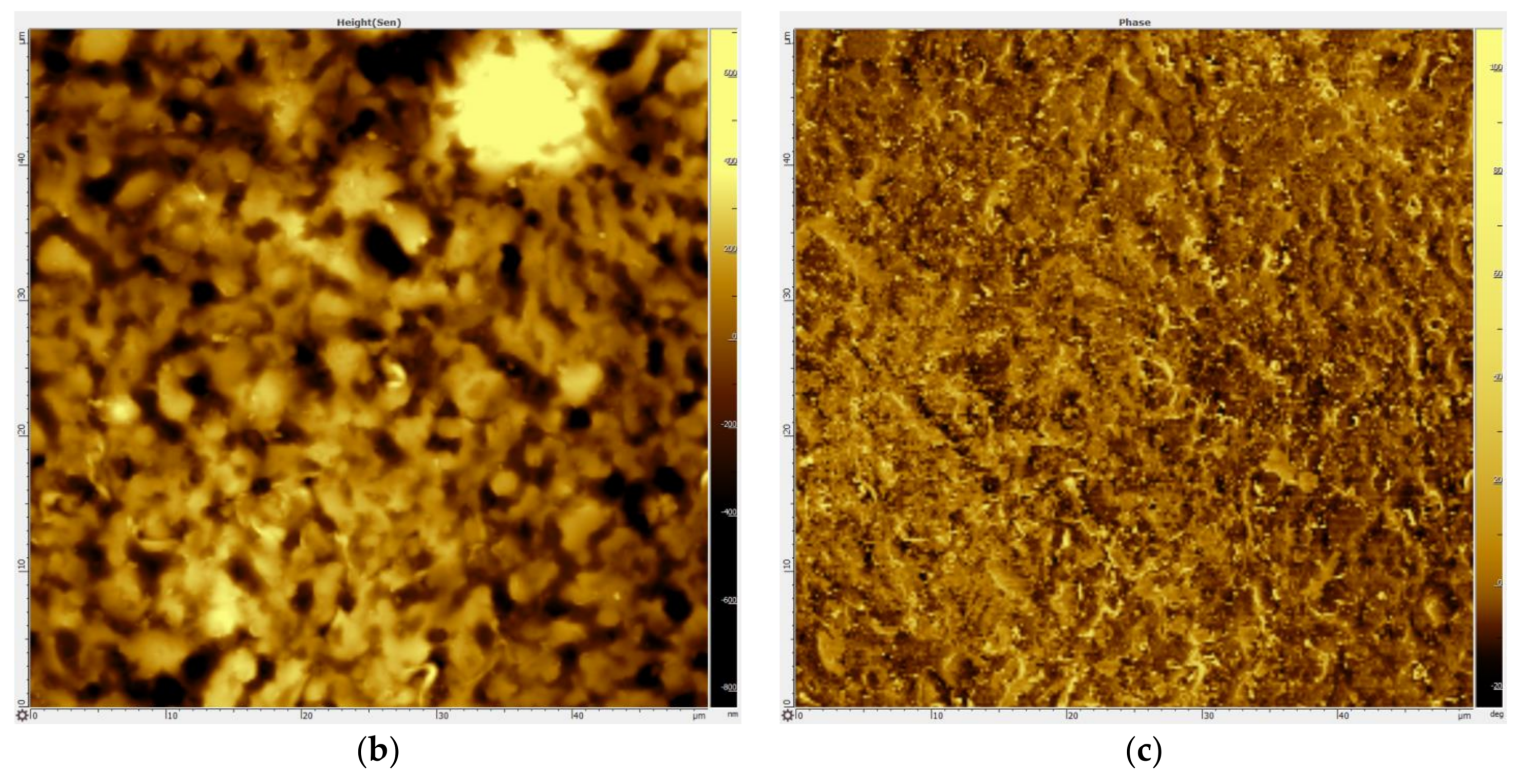
AFM-images of the surface of MF-4SC/halloysite+Pt membrane layer 2 ((**a**) clear polymer, 15 µm × 15 µm) and membrane layer 1 (modified polymer, 50 µm × 50 µm, (**b**) semi-contact mode; (**c**) phase-contrast mode).

TEM was used to confirm the position of platinum onto synthesized nanomaterials. Figure 1 demonstrates the presence of metallic nanoparticles and their clusters outside the halloysite nanotubes (over their outer surface). Two opposite surfaces of the bi-layer membrane having one layer modified with halloysite nanotubes and platinum nanoparticles obtained by AFM method is presented in Figure 2. Regular white circular dots (elevations) and small dark areas (valleys) in Figure 2a indicate minor defects appeared at pure membrane MF-4SC layer (without modifiers) contacted with glass during the drying procedure. Figure 2b,c shows the modified layer surface presented in semi-contact and phase-contrast mode of AFM, respectively. In both figures, we see woven polymer chains. White dots and slightly curved white “worms” in Figure 2c are halloysite nanotubes randomly and uniformly distributed in a polymer matrix.

### 2.3. Hybrid Membrane Synthesis

**Figure 3:**
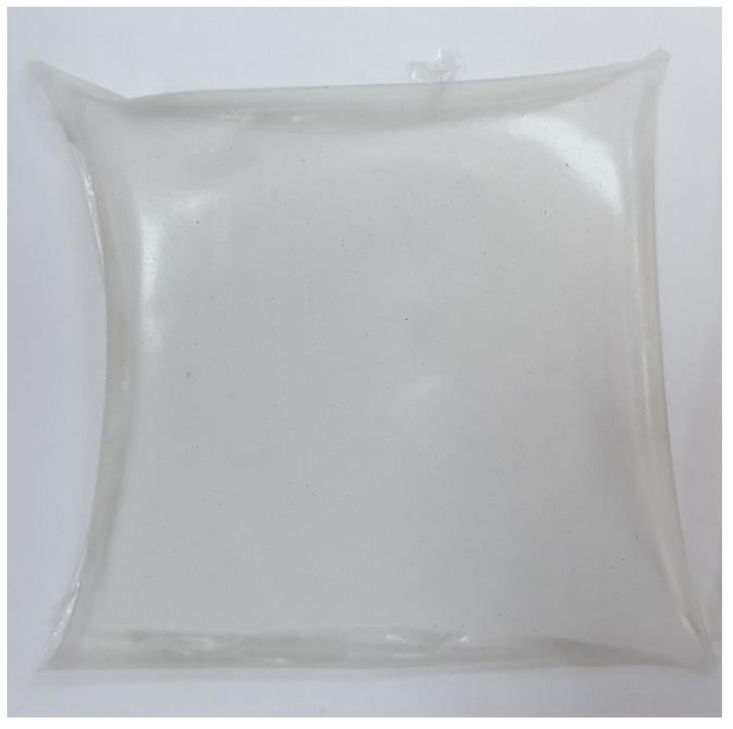
The bilayer membrane prepared by 2 steps method.

Bi-layer membranes were prepared by a novel approach that included two steps. First, to create thicker membrane layer (to be referred as 2-nd layer), the polymer solution was placed into a glass former and kept at least 2 h to ensure uniform distribution over the surface and removal of air bubbles. Then, the polymer solution was dried at a temperature 65 °C until completely solvent evaporation (about 1.5–2 h). The second step was coating of the 2-nd warmed up layer by the polymer solution with non- and modified nanotubes (to be referred as 1-st layer) by means of airbrush. Spraying was carrying out gradually to prevent dissolution of the 2-nd layer by new portions of the suspension solvent at a pressure 3 atm. Further, the 1-st membrane layer was dried at a temperature of 80 °C to remove residual solvent. After that, the film was neatly removed from the glass surface. The membrane was visually homogeneous over the entire area of both the sample surfaces (Figure 3). The light-grey membrane color resulted from the color of halloysite nanotubes modified by Pt. The ratio of the width of layers 2 to 1 was 4:1, and the total thickness of the obtained membranes was approximately 160–220 μm. The content of the halloysite nanotubes was 4 wt % of the 2-nd membrane layer. The content of the modifying metal was 2 wt % of the nanotubes’ mass. Such percentage of platinum was chosen based on previous research [18]. We conducted additional studies of the mechanical properties of synthesized hybrid membranes depending on the percentage of halloysite that confirmed the optimal value of halloysite nanotubes is equal to 4 weight percent (see Section 2.4). This value corresponds to the largest Young’s modulus and the tensile strain among the composite films doped with halloysite [19]. It means that such membranes have better mechanical characteristics. Three bi-layer membranes were prepared for investigations, and their composition is given in Table 1. 

Platinum nanoparticles (20–40 nm in diameter) were encapsulated on the outer surface of halloysite nanotubes. For simplicity, we will denote membranes as follows: No. 1: P/P + H + Pt; No. 2: P/P + H; No. 3: P + H/P + H + Pt.

**Figure 4:**
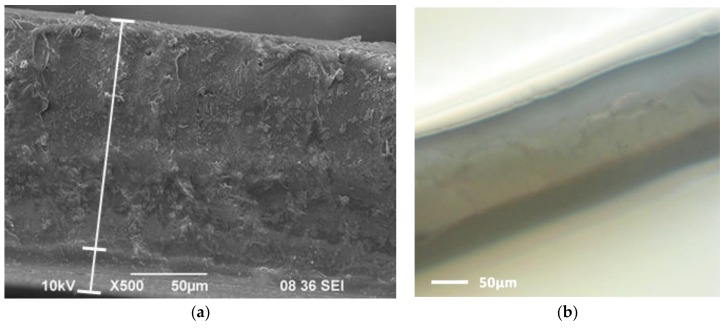
SEM micrography (**a**) and optical microscope image; (**b**) of the normal cross-section of the bi-layer membrane No. 1.

To confirm bi-layer structure of synthesized composites, we used micrography of the normal cut-off of No. 1 membrane (Figure 4a).

It is seen that there is a modified layer having a different structure (at the bottom of the film) which is approximately four times thinner than non-modified layer. The contrast of two membranes layers can be seen clearly in Figure 4b, which is a photo made using optical microscope.

### 2.4. Mechanical Testing

**Figure 5:**
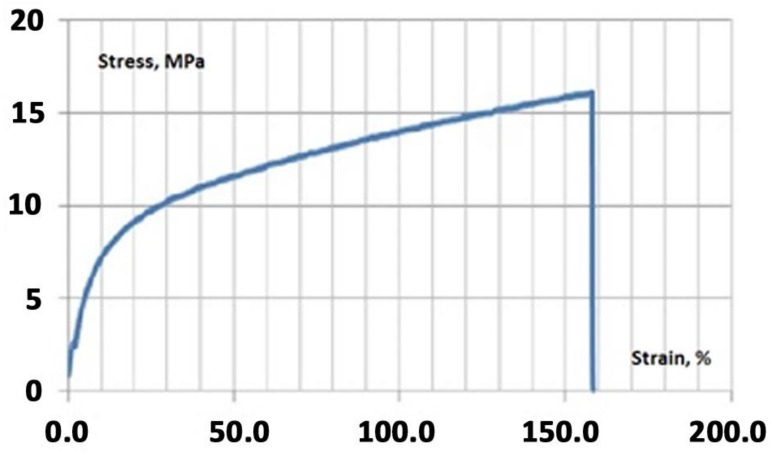
Typical stress–strain curve of the one-layer MF-4SC membrane.

To find the optimal content in view of the best mechanical properties we cast and tested several one-layer membranes with different percentages of halloysite nanotubes. Investigations of the mechanical characteristics of hybrid membranes were carried out using a TT-1100 tearing machine (Cheminstruments, Fairfield, OH, USA) at room temperature (25 °C). The traverse speed was 3.8 cm/min. Samples were pieces of films of rectangular shape about 90 mm long and about 10 mm wide. The initial distance between the clamps was 60–75 mm in dependence on the length of the film stripes. The modulus of elasticity was determined from the slope of the stress–strain curve close to the rectilinear section, with strain values not exceeding 5%. The stresses were calculated based on the initial cross-section of the sample. Typical stress-strain curve is shown in Figure 5.

**Figure 6:**
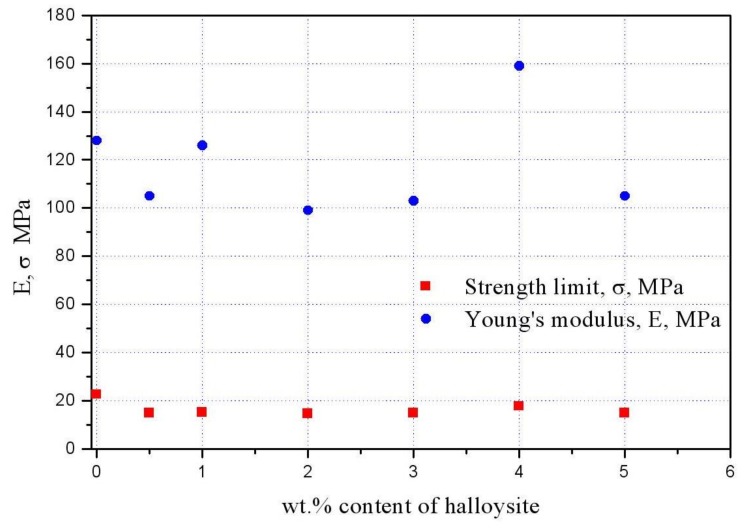
Dependence of elastic modulus (*E*) and strength limit (σ) on content of halloysite nanotubes in the film.

The curve has pronounced elastic and viscous plastic regions. The destruction of the film occurs on a fragile scenario with an elongation of about 150% at the time of destruction. Figure 6 presents dependences of elastic modulus (*E*) and strength limit (σ) on content of halloysite in the membrane. For comparison, low density polyethylene has Young’s modulus equal to *E* = 150–250 MPa. It can be seen from Figure 6 that the membrane with halloysite content of 4% by weight has the largest modulus of elasticity and strength limit. Therefore, in our studies, we used just such a percentage of the mineral.

## 3. Membrane Characterization

After synthesis, all membranes were washed with water and then equilibrated with NaCl solutions at the specified concentrations. Following this, their transport characteristics (integral diffusion permeability and current–voltage curves) were investigated with experimental methods described in [20]. 

### 3.1. Diffusion Permeability Measurements

**Figure 7:**
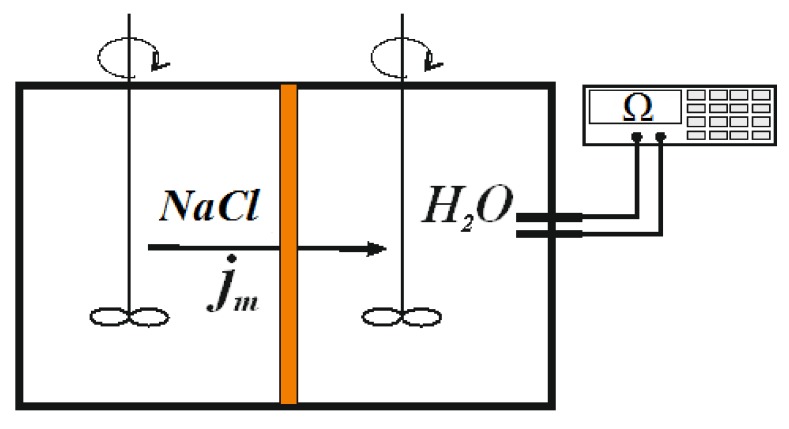
Two-chamber cell for measuring diffusion permeability.

Determination of the diffusion transmembrane flux density (*j*_m_) of electrolyte (NaCl) with concentration *C*_0_ and the integral coefficient of diffusion permeability (*P* = *j*_m_*h*/*C*_0_) of the membrane with total thickness *h* = *h*_1_ + *h*_2_ as a function of the orientation of the bi-layer membrane was carried out in a two-chamber cell equipped with platinized platinum electrodes and two magnetic stirrers based on the experimental determination of the diffusion rate of an electrolyte through a membrane into water by the conductometric method. Circular velocity of the stirring was equal to 200 rpm. Scheme of the diffusion cell is shown in Figure 7. All measurements were performed in solutions of sodium chloride in the concentration range 0.1–1.0 M and results are placed in Table 2. Subscript “s” means orientation of the first (modified and thinner) layer towards electrolyte solution (s-orientation) and subscript “w” means orientation of the first layer towards the chamber with pure water (w-orientation). 

### 3.2. Current–Voltage Curves Measurement

**Figure 8:**
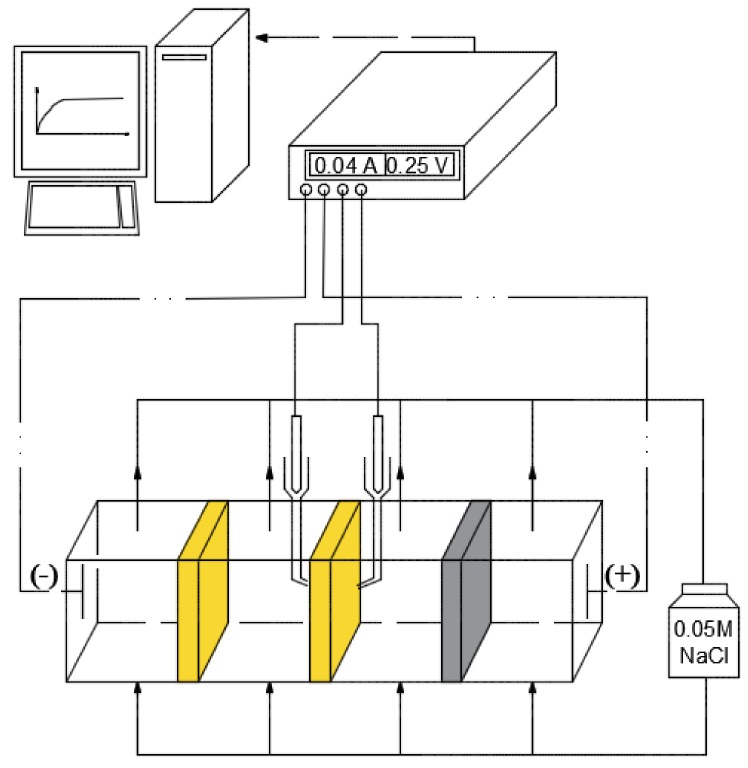
The scheme of current–voltage curve (CVC) measuring procedure.

The current–voltage curves (CVCs) of membranes were obtained in the galvanodynamic regime at scanning rate of 10^−4^ A/s in 0.05 M NaCl solution. Direct current was applied at a specified scan rate to the platinum polarizing electrodes using an Autolab PGSTAT302N potentiostat/galvanostat (Metrohm Autolab B.V., Utrecht, The Netherlands). The change of the potential difference across the membrane was recorded using Luggin–Haber capillaries connected with the membrane and the measuring silver/silver chloride electrodes (Figure 8). The measurements of CVCs for ion-exchange membranes occur under conditions of the laminar flow of solution with volume cross-flow velocity of the solution equal to 14 mL/min. 

The parameters of CVCs, such as the limiting current density *I*_lim_, the length of limiting current plateau Δ*U*, the slopes of the ohmic (Δ*I*/Δ*U*)_ohm_, limiting (Δ*I*/Δ*U*)_lim_, and overlimiting (Δ*I*/Δ*U*)_overlim_ parts, were found graphically from the CVCs using Microsoft Excel. During the study of the polarization behavior of the samples, several CVCs (up to 10) were measured at the same orientation of the membrane in the measuring cell, and we achieved high reproducibility of current–voltage curves. 

Note, that we observe the asymmetry of the CVC for samples No. 2 and 3 upon changing the membrane orientation of the modified and thinner layer towards anode for both bi-layer membranes. Membrane No. 1 did not show asymmetry of CVC within the experimental error. The calculated parameters of the CVC for all membranes under consideration are shown in Table 3. As the table shows, the deviation from the mean values of the CVC parameters, such as the slope of the CVC ohmic portion, the value of the limiting current density, *I*_lim_, and the length ∆*U* of the limiting current plateau, depend on the orientation of the membrane and its composition. The shortest plateau appears in the membrane No. 1 in the case of s-orientation (modified layer is faced to anode), and in membrane No. 3, in the case of w-orientation (modified layer is faced to cathode). The modification also leads to an increase in the limiting current density of all samples, but more pronounced (by approximately 17 percent) for Nos. 2 and 3. Sample No. 1 has highest slope of the overlimiting part of the CVC, and this means that for the same values of electrical potential drop across the bi-layer membranes, it is possible to attain high current densities under overlimiting current mode that occurs earlier than for the membrane consisted of thicker layer doped only by halloysite and thinner layer modified by halloysite and platinum (sample No. 3).

## 4. Theory

### 4.1. Extraction of Physicochemical Parameters for Membrane Layers

To calculate the physicochemical parameters of the layers, we used the formulas for the integral coefficient of diffusion permeability *P* obtained in our work [21] for the case of the practical absence of asymmetry of this coefficient in the case of differing characteristics of the layers. The use of the bi-layer model [16,21] allowed us earlier to explain the absence of an asymmetry of the diffusion permeability in layered composites with a fixed thickness of the polyaniline layer. In this case, between the physicochemical and geometric parameters, two relations must be fulfilled: $\frac{D_{m1}h_{2}\gamma_{m2}}{D_{m2}h_{1}\gamma_{m1}}=1,\;\bar{\rho }\equiv \rho_{2}\gamma_{m2}=\rho_{1}\gamma_{m1}$ (that is, the effective exchange capacity $\bar{\rho }$ is constant throughout the thickness of the membrane). Here, $\rho_{1},\;\rho_{2}$—exchange capacities of the layers, $\gamma_{m1},\;\gamma_{m2}$—coefficients of the equilibrium distribution of the electrolyte molecules in these layers of thickness $h_{1}$ and $h_{2}$. Only in this case, diffusion permeability remains the same with a change in the orientation of the membrane in the measuring cell, despite the different exchange capacities of the layers, i.e.,

(1) P≡Ps=C0(Dm1γm1)(1+H)(ρ¯)2+4C02+|ρ¯|≡Pw=C0(Dm2γm2)(1+1/H)(ρ¯)2+4C02+|ρ¯|,

where $H=h_{2}/h_{1}$—relative thickness of thicker layer 2. It follows from the above condition $\frac{D_{m1}h_{2}\gamma_{2}}{D_{m2}h_{1}\gamma_{1}}=1$ that the diffusion coefficients of the electrolyte molecules in the membrane layers are linearly related:

(2) Dm2γm2=H⋅Dm1γm1.

Since the values of *H* = 4 were constant for all three bilayer membranes, the magnitudes of the effective exchange capacity $\bar{\rho }$ and the diffusion coefficients of the electrolyte molecule Dm1γm1 and Dm2γm2 in the layers were calculated from the Formula (1) and the concentration data *P_s_*/*P_w_* of Table 2 by the least squares method using the Mathematica 11 computing system. The results of the calculations are given in Table 4. Note that the calculated data for the parameter Dm2γm2 are consistent with the previously obtained value of 43 μm^2^/s in an independent experiment for the non-modified MF-4SC membrane [22]. As can be seen from Table 4, condition (2) is observed with good accuracy—the effective diffusion coefficient of the electrolyte molecule in the thin layer 1 is lower by approximately 4 times than in the thick layer 2, that could indirectly confirm the compaction of the structure of the modified layer due to the addition of halloysite nanotubes and platinum.

### 4.2. Calculation of Limiting Currents for Different Membrane Orientations

To calculate, a priori, the limiting current density *I*_lim_ (one of the basic characteristics of CVC) we can apply the model of bi-layer cation-exchange membrane developed recently in [17] basing on parameters extracted from diffusion experiments (Table 4). When the membrane is oriented by a modified (thinner) layer 1 to the anode, we find an appropriate value of $I_{\lim}^{s}$ as a solution of the set of two implicit algebraic equations:

(3) $$\begin{array}{c} \sqrt{{\bar{\xi }}^{2}+{\bar{\sigma }}_{1}^{2}-{\bar{\sigma }}_{2}^{2}}+\frac{2}{\Delta }\frac{{\bar{v}}_{m_{1}}}{1+H}={\bar{\sigma }}_{1}+\frac{i_{\lim}^{s}\Delta }{2}{\bar{\sigma }}_{1}\ln\frac{{\bar{\sigma }}_{1}(1-\frac{i_{\lim}^{s}\Delta }{2})}{\sqrt{{\bar{\xi }}^{2}+{\bar{\sigma }}_{1}^{2}-{\bar{\sigma }}_{2}^{2}}-\frac{i_{\lim}^{s}\Delta }{2}{\bar{\sigma }}_{1}}\\ \bar{\xi }-\frac{2}{\Delta }\frac{{\bar{v}}_{m_{2}}H}{1+H}=\sqrt{{\bar{\sigma }}_{2}^{2}+16}+\frac{i_{\lim}^{s}\Delta }{2}{\bar{\sigma }}_{2}\ln\frac{\sqrt{{\bar{\sigma }}_{2}^{2}+16}-\frac{i_{\lim}^{s}\Delta }{2}{\bar{\sigma }}_{2}}{\bar{\xi }-\frac{i_{\lim}^{s}\Delta }{2}{\bar{\sigma }}_{2}} \end{array},$$

When the membrane is faced by a modified layer to the cathode, we find an appropriate value of $I_{\lim}^{w}$ as a solution of the system of two other implicit algebraic equations:

(4) $$\begin{array}{c} \sqrt{{\bar{\xi }}^{2}+{\bar{\sigma }}_{1}^{2}-{\bar{\sigma }}_{2}^{2}}+\frac{2}{\Delta }\frac{{\bar{v}}_{m_{1}}}{1+H}=\sqrt{{\bar{\sigma }}_{1}^{2}+16}+\frac{i_{\lim}^{s}\Delta }{2}{\bar{\sigma }}_{1}\ln\frac{\sqrt{{\bar{\sigma }}_{2}^{2}+16}-\frac{i_{\lim}^{s}\Delta }{2}{\bar{\sigma }}_{1}}{\sqrt{{\bar{\xi }}^{2}+{\bar{\sigma }}_{1}^{2}-{\bar{\sigma }}_{2}^{2}}-\frac{i_{\lim}^{s}\Delta }{2}{\bar{\sigma }}_{1}}\\ \bar{\xi }-\frac{2}{\Delta }\frac{{\bar{v}}_{m_{2}}H}{1+H}={\bar{\sigma }}_{2}+\frac{i_{\lim}^{s}\Delta }{2}{\bar{\sigma }}_{2}\ln\frac{{\bar{\sigma }}_{1}(1-\frac{i_{\lim}^{s}\Delta }{2})}{\bar{\xi }-\frac{i_{\lim}^{s}\Delta }{2}{\bar{\sigma }}_{2}} \end{array},$$

Here, $H=\frac{h_{1}}{h_{2}}$, $\Delta =\frac{\delta }{h_{1}+h_{2}}$, ${\bar{v}}_{m1,m2}=\frac{D\gamma_{m1,m2}}{D_{m1,m2}}$, ${\bar{\sigma }}_{1,2}={\bar{\rho }}_{1,2}/C_{0}$, $i_{\lim}^{s,w}=\frac{i_{\lim}^{s,w}(h_{1}+h_{2})}{C_{0}FD}$—dimensionless values of the limiting current densities, *δ*—thickness of diffusion layers adjacent to both membrane surfaces, which depends on stirring conditions, *D*—diffusion coefficient of the electrolyte molecule in dilute solution, *F*—the Faraday constant, and $\bar{\xi }$—dimensionless normalized sum of ions concentration on the right side of the interface between two layers (auxiliary parameter) [15,17]. To draw the full CVC (up to the overlimiting part) one may apply formulas also derived in [17].

## 5. Results and Discussion

**Figure 9:**
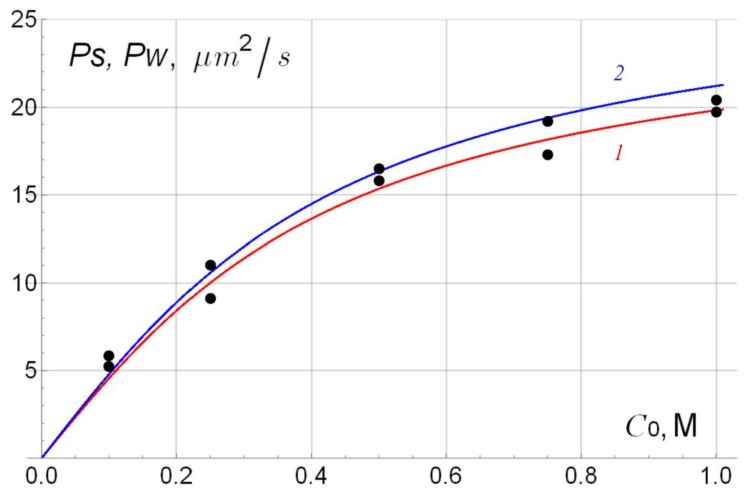
Concentration dependences of *P_s_* (*1*) and *P_w_* (*2*) for membrane No. 2 (according to Table 1 and Equation (1)).

In Figure 9, as an illustration, the experimental and theoretical dependences of the integral coefficients of the diffusion permeability *P*_s_ and *P*_w_ in the case of their greatest discrepancy (membrane No. 2), on average equal to 1.16 μm^2^/s per measured point, are given. In the second place, according to this discrepancy, there is membrane No. 1 (0.97 μm^2^/s), in the third place, membrane No. 3 (0.59 μm^2^/s). Note that membranes No. 1 and No. 2 have the same thicker layer 2 of pure polymer, synthesized by casting, so we used the same parameters $\rho_{2}\gamma_{m2}$ and $D_{m2}/\gamma_{m2}$ of this layer obtained in calculating the dependence of the diffusion permeability on the concentration for membrane No. 1 (see the penultimate column of Table 4). For all membranes, the theoretical dependence of *P_w_* on the concentration (the upper curve in Figure 9) is somewhat higher than the dependence of *P_s_* (the lower curve in Figure 9), which is a consequence [16] of the inequality $\rho_{2}\gamma_{m2}>\rho_{1}\gamma_{m1}$ (the effective exchange capacity of the thicker layer 2 is higher than that of the modified layer 1).

Exchange capacities of three hybrid monolayer membranes were found in independent experiments to be equal to 1.08, 1.15, and 1.22 mole/l for pristine MF-4SC, MF-4SC doped with 4 wt % of halloysite nanotubes, and MF-4SC doped with 4 wt % of halloysite nanotubes encapsulated by platinum nanoparticles, respectively. After this, using the data in Table 4, it became possible to determine the intrinsic coefficients of the equilibrium distribution and diffusion of the electrolyte molecules in the layers of the membrane (Table 5). Despite the fact that membranes No. 1 and 3 have a modified layer of the same composition, this layer of membrane No. 3 is denser (has a lower diffusion coefficient $D_{m1}$ of the electrolyte) and has stronger positive adsorption of the electrolyte molecules, i.e., has lower coefficient $\gamma_{m1}$. This, in particular, might be due to the fact that the modified layer 1 in these membranes is applied by airbrush to different substrates (thick layers 2), and the thickness of membrane No. 3 is one third smaller.

In order to calculate the densities of the limiting currents from Equations (3) and (4) for different orientations of the membrane, one can use Table 4 and Table 5. In our case, *D* = *D*_NaCl_ = 1622 µm^2^/s, so ${\bar{\nu }}_{m1}=153.9;\;155.5;\;192.6$ and ${\bar{\nu }}_{m2}=36.1;\;40.1;\;45.2$, consequently for membranes No. 1–3. Both systems of Equations (3) and (4) should be solved numerically. To obtain the dimensional values of voltage U and current densities $I_{\lim}^{s,w}$, their dimensionless analogs must be multiplied correspondingly by RT/F=25.67 mV and by $C_{0}FD/\left(h_{1}+h_{2}\right)=35.41$, 43.23 and 47.14 A/m^2^ consequentially for 1-st, 2-nd, and 3-rd membranes. The system of Equations (3) and (4) can be regarded as a tool to find the thickness, *δ*, of diffusion layers because we know the limiting current densities from experiments (Table 3). We calculated the thicknesses of the diffusion layers for all hybrid membranes using specially created program for Mathematica 11, and the results, depending on the orientation of the membranes with respect to the applied electric field, were placed in Table 6.

**Figure 10:**
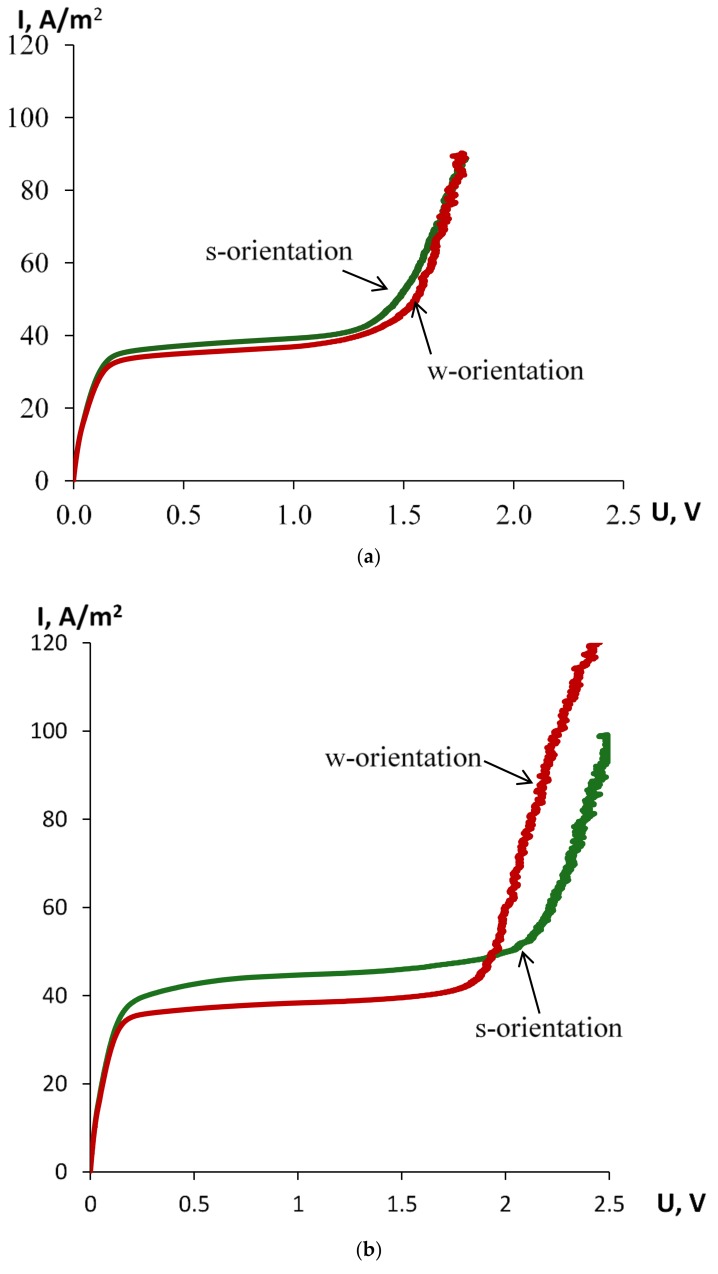

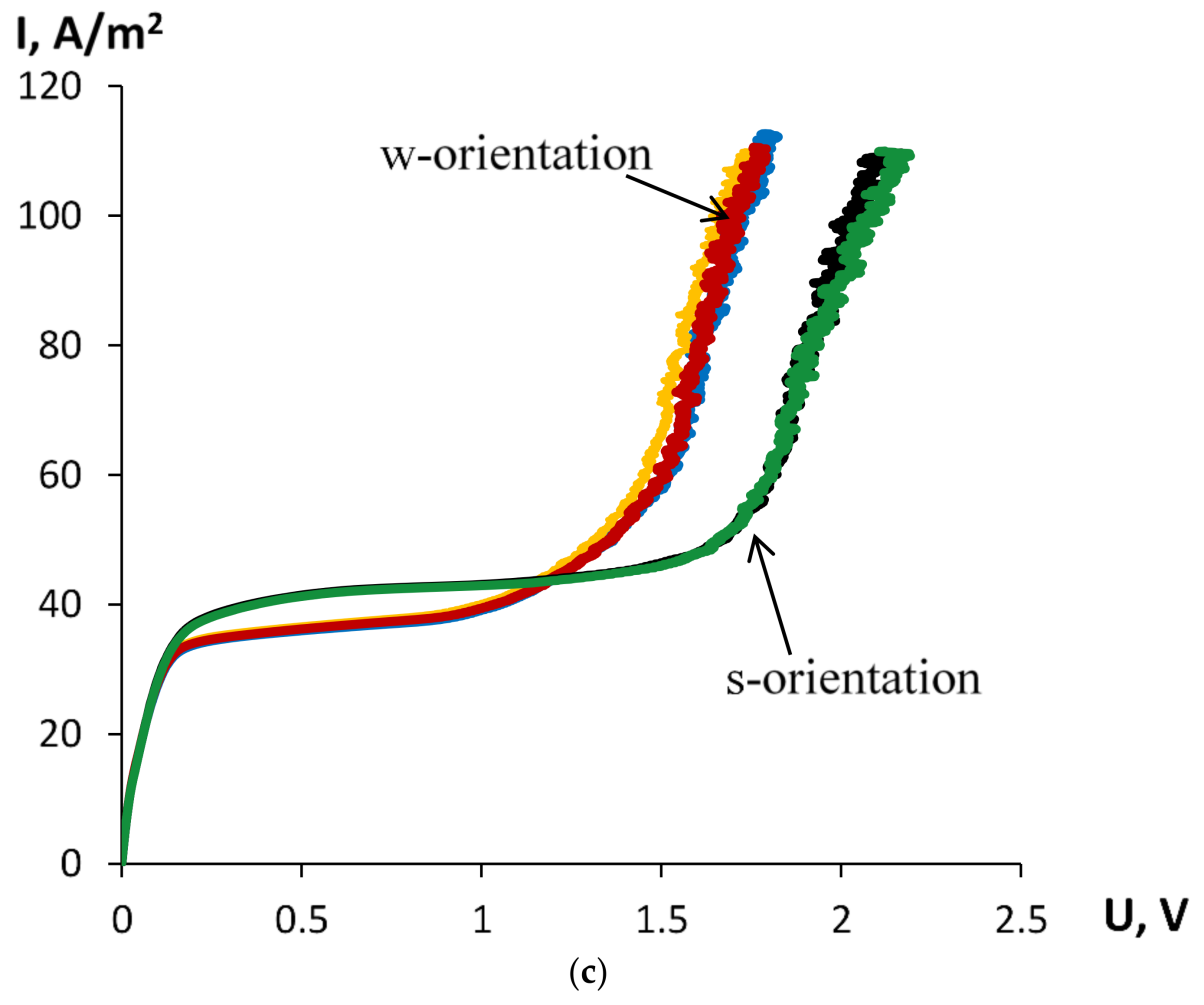
(**a**) Experimental CVCs of hybrid bi-layer membrane No. 1 (P/P + H + Pt) under different orientation in the measuring cell; (**b**) Experimental CVCs of hybrid bi-layer membrane No. 2 (P/P + H) under different orientation in the measuring cell; (**c**) Experimental CVCs of hybrid bi-layer membrane No. 3 (P + H/P + H + Pt) under different orientation in the measuring cell.

Figure 10 presents experimental CVCs of all three hybrid membranes for their two orientations inside measuring cell. From Table 6 and Figure 10, it follows that dimensionless values of $i_{\lim}^{s,w}$ fluctuate around unity. It is interesting to compare the results obtained with the case of a perm-selective (ideal) cation-exchange membrane, when its current–voltage characteristic is given by the simple equation

(5) $$I=\frac{FD_{+}Z_{+}C_{0+}}{\delta }\left(1+\frac{Z_{+}}{Z_{-}}\right)\left(1-\exp\left(-Z_{-}U\right)\right),$$

and the limiting current density can be found from (5) applying the classical formula derived by Isaac Rubinstein:

(6) $$I_{\lim}=\underset{U\to \infty }{\lim}I=\frac{FD_{+}Z_{+}C_{0+}}{\delta }\left(1+\frac{Z_{+}}{Z_{-}}\right)\equiv \frac{FD_{+}C_{0}}{\delta }\left(1+\frac{Z_{+}}{Z_{-}}\right)$$

In the case under consideration, we have $Z_{+}=Z_{-}=1$ and $D_{+}=D_{Na^{+}}=1350\;{\mu m}^{2}/s$. Using (6), we calculated values of $\delta_{\mathrm{ideal}}=\frac{2FD_{+}C_{0}}{I_{\lim}}$, and put them after slash into third and fifth column of Table 5. Analyzing the data from Table 5, we see that there are practically precise relations between the thicknesses of diffusion layer for perm-selective and real membranes, namely $\delta_{s}\approx 1.27\delta_{\mathrm{ideal}}$ and $\delta_{w}\approx 1.54\delta_{\mathrm{ideal}}$. The corresponding coefficients in the given ratios depend on the properties of the surface facing the anode, and the degree of imperfection of the membrane. In all cases, the thickness of the diffusion layer is less when the modified layer is oriented into the desalting cell (s-orientation). This can be explained by the fact that the modified surface is rougher (Figure 2b,c), and the surface of the thicker layer (Figure 2a) is smooth, as it was turned to the bottom glass of the Petri dish during the membrane casting. The rough surface forms vortices that partially destroy the diffusion layer [23]. In addition, due to the presence of halloysite nanotubes on the modified surface, it has a mosaic charge structure, i.e., has alternating charged and uncharged areas, which also contributes to the formation of electroconvective vortices [24].

Figure 10 shows that less asymmetry of the CVC is observed for the first membrane (Figure 10a), and the largest for the second membrane (Figure 10b), which also has more pronounced diffusion permeability asymmetry (Figure 9). Figure 10b,c illustrate, in comparison, that introduction of 4 wt % halloysite nanotubes in one layer of perfluorinated matrix MF-4SC increases the limiting current density and leads to asymmetry of CVC, while the addition of halloysite nanotubes functionalized with platinum partly compensates for the effect of halloysite adding. The addition of platinum nanoparticles to the external surface of halloysite nanotubes leads to a reduction in the plateau of the limiting current by 25%–50%, which is more significant in the case of the w-orientation of the membrane (a modified layer is turned to the cathode). The shortening of the plateau of the limiting current can be connected with the catalytic action of platinum on the process of water splitting, which leads to the appearance of additional charge carriers—protons and hydroxyl ions. In order to illustrate the high reproducibility of the results, Figure 10c shows 2–3 CVCs measured for each orientation of the membrane. We see a practical coincidence of these curves, even in the overlimiting regime.

## 6. Conclusions

This study of the transport characteristics of three bi-layer hybrid nanocomposites synthesized on the base of perfluorinated membrane MF-4SC (Russian analogue of Nafion^®^-117), and their modifications with halloysite nanotubes and platinum nanoparticles, showed that two of them possess the noticeable asymmetry of the current–voltage curve (CVC) under changing their orientation towards applied electric field, despite the practical absence of asymmetry of diffusion permeability. It was shown that introduction of 4 wt % halloysite nanotubes in one layer of perfluorinated matrix MF-4SC increases the limiting current density, and leads to asymmetry of CVC, while the addition of halloysite nanotubes functionalized with platinum partly compensates for the effect of halloysite addition. At the same time, the analysis of the CVC parameters revealed the catalytic action of platinum nanoparticles on the process of water splitting. This makes it promising to predict the effective use of hybrid membranes based on MF-4SC and halloysite nanotubes with platinum nanoparticles, not only as separating films in fuel cells and electromembrane devices, but also as promising catalytic systems. The asymmetry phenomenon for the current–voltage characteristics and symmetry for the diffusion permeability coefficient were explained in the framework of the “fine-porous model”, which was extended for bi-layer membranes. A special procedure to calculate the real values of the diffusion layers thickness and the limiting current density was proposed. Due to asymmetry effects of the current–voltage curves of bi-layer hybrid membranes on the base of MF-4SC, halloysite nanotubes, and platinum nanoparticles, it is prospective to assemble membrane switches (membrane relays or diodes) with predictable transport properties, founding upon the theory developed here. 

## Figures and Tables

**Table 1:** Composition of synthesized bi-layer membranes.

Bi-Layer Membrane	Thickness, *h*, μm	Thin Layer (1)	Thick Layer (2)
No. 1	221	MF-4SC modified with 4 wt % of halloysite nanotubes encapsulated by 2 wt % platinum nanoparticles	Pristine MF-4SC membrane
No. 2	181	MF-4SC modified with 4 wt % of halloysite nanotubes	Pristine MF-4SC membrane
No. 3	166	MF-4SC modified with 4 wt % of halloysite nanotubes encapsulated by 2 wt % platinum nanoparticles	MF-4SC membrane modified with 4 wt % of halloysite nanotubes

**Table 2:** Experimental diffusion permeability in NaCl solution for three bi-layer hybrid membranes based on MF-4SC.

*С*_0_ M	*P*_s_/*P*_w_, μm^2^/s, P/P + H + Pt Bi-Layer Membrane No. 1	*P*_s_/*P*_w_, μm^2^/s, P/P + H Bi-Layer Membrane No. 2	*P*_s_/*P*_w_, μm^2^/s, P + H/P + H + Pt Bi-Layer Membrane No. 3
0.1	5.92/6.30	5.84/5.23	4.38/5.13
0.25	9.73/10.9	9.12/11.0	8.69/8.40
0.5	15.5/14.4	15.8/16.5	13.8/13.5
0.75	17.8/19.7	17.3/19.2	14.7/15.5
1.0	20.8/21.9	20.4/19.7	16.9/17.7

**Table 3:** Parameters of the CVC of composite bi-layer membranes Nos. 1–3 from Table 1.

Orientation towards Anode by	*I*_lim_, A/m^2^	Δ*U*, V	Slope of the Ohmic Part (Δ*I*/Δ*U*)_ohm_	Slope of the Limiting Part (Δ*I*/Δ*U*)_lim_	Slope of the Overlimiting Part (Δ*I*/Δ*U*)_overlim_
**Membrane No. 1**					
thinner layer 1	35.79 ± 0.49	1.34 ± 0.01	347.0 ± 0.1	4.51 ± 0.01	158.7 ± 16.8
thicker layer 2	33.57±1.32	1.42 ± 0.01	383.0 ± 0.2	3.94 ± 0.12	194.5 ± 5.2
**Membrane No. 2**					
thinner layer 1	42.02 ± 0.22	2.02 ± 0.01	337.6 ± 0.1	2.70 ± 0.01	135.2 ± 4.3
thicker layer 2	35.99 ± 0.02	1.75 ± 0.01	314.6 ± 0.1	2.52 ± 0.00	153.2 ± 10.2
**Membrane No. 3**					
thinner layer 1	40.40 ± 0.16	1.57 ± 0.03	317.7 ± 0.1	3.14 ± 0.00	148.2 ± 21.5
thicker layer 2	34.38 ± 0.54	1.27 ± 0.03	318.7 ± 0.1	4.85 ± 0.00	180.7 ± 0.1

**Table 4:** Calculated physicochemical parameters of the hybrid bi-layer membranes.

Bi-Layer Membrane	*ρ*_1_*γ_m_*_1_, M	*ρ*_2_*γ_m_*_2_, M	*D_m_*_1_/*γ_m_*_1_, μm^2^/s	*D_m_*_2_/*γ_m_*_2_, μm^2^/s
**No. 1** (P/P + H + Pt), *h* = 221 μm	0.545	0.569	10.54	44.94
**No. 2** (P/P + H), *h* = 181 μm	0.555	0.569	10.43	44.94
**No. 3** (P + H/P + H + Pt), *h* = 166 μm	0.480	0.527	8.42	35.88

**Table 5:** Physicochemical parameters of the hybrid bi-layer membranes.

Bi-Layer Membrane	*ρ*_1_, mole/L	*ρ*_2_, mole/L	*D_m_*_1_, μm^2^/s	*D_m_*_2_, μm^2^/s	*γ_m_*_1_	*γ_m_*_2_
No. 1 (P/P + H + Pt), *h* = 221 μm	1.22	1.08	4.71	23.68	0.447	0.527
No. 2 (P/P + H), *h* = 181 μm	1.15	1.08	5.04	23.68	0.483	0.527
No. 3 (P + H/P + H + Pt), *h* = 166 μm	1.22	1.15	3.31	16.44	0.393	0.458

**Table 6:** Calculated thicknesses *δ* of diffusion layers of the hybrid bi-layer membranes.

Bi-Layer Membrane	*h*, μm	*δ_s_*/*δ*_ideal_, μm	$i_{\lim}^{s}$	*δ_w_*/*δ*_ideal_, μm	$i_{\lim}^{w}$
		*s-Orientation*		*w-Orientation*	
**No. 1** (P/P + H + Pt),	221	464/365 = 1.27	1.011 ± 0.014	594/388 = 1.53	0.948 ± 0.037
**No. 2** (P/P + H),	181	394/310 = 1.27	0.972 ± 0.005	558/361 = 1.55	0.833 ± 0.001
**No. 3** (P + H/P + H + Pt),	166	415/323 = 1.28	0.857 ± 0.003	586/380 = 1.54	0.729 ± 0.012

## Notation List

*Latin*	
*C*_0_, *C*_0+_, *C*_0__–_	electrolyte and ion concentrations
*D*	diffusion coefficient of the electrolyte molecule in dilute solution
$D_{mi}$	diffusion coefficients of the electrolyte molecule inside *i*-layer
*F*	Faraday constant
*h_i_*	thickness of *i*-layer
*h* = *h*_1_ + *h*_2_	thickness of the membrane
*H* = *h*_2_/*h*_1_	ratio of membrane layer thicknesses
*I*, *i*	dimension and dimensionless current density
$I_{\lim}^{s,w}$ and $i_{\lim}^{s,w}=\frac{I_{\lim}^{s,w}\left(h_{1}+h_{2}\right)}{C_{0}FD}$	dimension and dimensionless values of the limiting current densities
*j_m_*	transmembrane flux density of electrolyte
*P*	coefficient of the integral diffusion permeability
*R*	universal gas constant
*T*	absolute temperature
*U*, *u*	dimension and dimensionless voltage
*Greek*	
*δ*	thickness of diffusion layers adjacent to both membrane surfaces
Δ = *δ*/(*h*_1_ + *h*_2_)	dimensionless thickness of diffusion layers
Δ*U*	voltage difference
Δ*I*	current density difference
$\gamma_{mi},\;\gamma_{mi\pm }$	equilibrium distribution coefficients of the electrolyte molecule and individual ions inside membrane *i*-layer
${\bar{\nu }}_{m1,m2}=\frac{D\gamma_{m1,m2}}{D_{m1,m2}}$	dimensionless physicochemical parameters
$\rho_{i},\;\sigma_{i}$	dimension and dimensionless *i*-layer exchange capacity
${\bar{\sigma }}_{i}=\;\sigma_{i}\gamma_{mi}$	effective dimensionless *i*-layer exchange capacity
$\bar{\xi }$	dimensionless normalized sum of ions concentration on the right side of the interface between two membrane layers
*Superscripts*	
*s*, *w*	related to a membrane oriented by modified layer to solution and water, respectively
*Subscripts*	
*i*	related to membrane layer number *i*
*m*	related to a membrane
*s*, *w*	related to a membrane oriented by modified (1-st) layer to solution (anode) and water (cathode), respectively
*lim*	related to the limiting current regime
*ohm*	related to the ohmic part of CVC
*overlim*	related to the overlimiting current regime
